# Implicit Attitude Toward Caregiving: The Moderating Role of Adult Attachment Styles

**DOI:** 10.3389/fpsyg.2015.01906

**Published:** 2016-01-07

**Authors:** Pietro De Carli, Angela Tagini, Diego Sarracino, Alessandra Santona, Laura Parolin

**Affiliations:** Department of Psychology, University of Milano-BicoccaMilan, Italy

**Keywords:** adult attachment, implicit measure, caregiving, intergenerational transmission, internal working model

## Abstract

Attachment and caregiving are separate motivational systems that share the common evolutionary purpose of favoring child security. In the goal of studying the processes underlying the transmission of attachment styles, this study focused on the role of adult attachment styles in shaping preferences toward particular styles of caregiving. We hypothesized a correspondence between attachment and caregiving styles: we expect an individual to show a preference for a caregiving behavior coherent with his/her own attachment style, in order to increase the chance of passing it on to offspring. We activated different representations of specific caregiving modalities in females, by using three videos in which mothers with different Adult Attachment states of mind played with their infants. Participants' facial expressions while watching were recorded and analyzed with FaceReader software. After each video, participants' attitudes toward the category “mother” were measured, both explicitly (semantic differential) and implicitly (single target-implicit association task, ST-IAT). Participants' adult attachment styles (experiences in close relationships revised) predicted attitudes scores, but only when measured implicitly. Participants scored higher on the ST-IAT after watching a video coherent with their attachment style. No effect was found on the facial expressions of disgust. These findings suggest a role of adult attachment styles in shaping implicit attitudes related to the caregiving system.

## Adult attachment styles and implicit attitudes toward caregiving styles

### Attachment theory

In the first formulations of attachment theory, Bowlby (1969/1982, 1980) postulated that children's need of their caregivers, in terms of proximity seeking, was indispensable for evolutionary adaptation. Children rely on caregivers for safety and resources, but attachment bonds have far-reaching implications since they are involved in shaping development. As Bowlby (1969/1982, p. 64) stated, “Not a single feature of a species' morphology, physiology, or behavior can be understood or even discussed intelligently except in relation to that species' environment of evolutionary adaptness (EEA).” This is consistent with the idea that less than optimal developments should be conceived as a sort of “over-adaptation” to a maladaptive environment rather than as mere failures in adaptation.

The evolutionary pressure that fosters mother-infant bonding lies at the core of adult social behaviors. Baumeister and Leary (1995) highlighted the benefits of forming and maintaining social bonds in terms of survival and reproduction, proposing that the need for interpersonal attachments constitutes a fundamental human motivation. Bowlby introduced the concept of internal working models (IWM), whose function is “to simulate happenings in the real world, thereby enabling the individual to plan his behavior with all the advantages of insight and foresight” (Bowlby, 1988). The IWMs are expectations aimed at the preservation of self-regulation and positive affect even in adverse environmental conditions (Sroufe and Jacobvitz, 1989; Schore, 1994). Main et al. (1985) went beyond the behavioral perspective by introducing a representational framework, in which IWMs are considered to be “a set of conscious and/or unconscious rules,” organizing attachment-related memories, emotions and thought processes. (p. 66).

### Social cognition of attachment

Westen (1991) proposed that attachment studies are one of the most productive fields in which psychodynamic and cognitive studies can be integrated. In his view, representations that underlie attachment-related processes constitute a key element in linking these two perspectives. In fact, Bowlby always underlined the primary role of beliefs and cognitive schemata in orienting attachment behaviors and expectations, but it is only after the reconceptualization of attachment theory in representational terms that social cognitive models and methods have effectively been implemented in this field (Bartholomew and Horowitz, 1991; Brennan et al., 1998; Brennan and Shaver, 2002; Mikulincer et al., 2005; Shaver and Mikulincer, 2013).

From a social cognition perspective, Baldwin et al. (1996) found that accessible memories of satisfying or unsatisfying attachment experiences play a role in shaping the perception of relationships. Moreover, they showed that mental schemas of attachment can have different accessibility depending on different contexts, in a similar way to the majority of cognitive structures. In particular, they asked the participant to visualize different kinds of relationships and this led them to react in different ways to interpersonal information and to show increased attraction toward potential dating partners with the same attachment orientation. This helped to improve our understanding of the IWMs moving forward from a definition based on personality traits to a more complex perspective based on a hierarchical structure of relationship specific attachment orientations (Bretherton, 1987, 1999). In the same perspective, Bartz and Lydon (2004) found the effect of manipulating close relationships on the working self-concept, in particular on agency and communion. Security priming of attachment leads to positive relationship expectations and affect (Rowe and Carnelley, 2003; Carnelley and Rowe, 2007; Gillath et al., 2008) and the effect seems stable across the life span and for different domains of social information processing (Dykas and Cassidy, 2011).

A social cognition perspective has been applied also to parenting behaviors. Atkinson et al. (2009) studied the interplay between emotional cognitive processes (selective attention in the Stroop task) and disorganized attachment in mothers and their children. They found an interesting interacting role of both attachment and loss of controlled attention that they interpret as a process of “threat tags.” They conclude that IWMs role in risk for psychopathology can be fully appreciated only if research tries to explore different levels of analysis of attachment representations (cognitive and affective). In fact, Okagaki and Bingham (2005) had tried to drive the attention of researchers to the relevance of a better comprehension of the relation between social cognition and behavior in order to develop effective parent intervention programs. For instance, parental stress seems to be associated to both anxiety and avoidance of attachment, because of the difficulties they imply in coping with distress, but in different ways: more avoidant women attribute negative distress to a characteristic of the baby and not situational factors; more anxious women make more mistakes in recognizing fear and attribute distress to physical factors, then they could show an out of sync response to the babies' distress signs (Leerkes and Siepak, 2006; for a complete review of a social cognition approach to parenting processes and behaviors, see: Jones et al., 2015a,b).

### Implicit activation of attachment representations

Attachment research studied the role of implicit activation of attachment representations (Gawronski and Payne, 2011), starting from Bowlby's idea of the importance of automatic attachment schemata. For instance, there is evidence of individual differences in expressing avoidance or hypervigilance with respect to attachment threats: fearful avoidant individuals are in fact characterized by cognitive avoidance of all highly emotional stimuli (Dewitte et al., 2007). Baldwin et al. (1993) as well as Mikulincer (1998) presented priming sentences like “If I trust my partner my partner will…” (this example manipulates the variable trust). Subsequently, a word non-word task with attachment related words (representing good or negative outcomes) as priming cues was administered. In general, insecure individuals were quicker in the negative outcome condition while secure were quicker in the good outcome condition. This result can be interpreted as insecure participants expecting less supportive partners in stressful conditions: for them the negative outcome is more naturally consequent to angry feelings.

One of the most widely-used paradigms to study the automatic activation of representations is the implicit association task (IAT; Greenwald et al., 1998, 2003), which assesses implicit dimensions of psychological constructs such as attitudes and stereotypes (for a description, see the Materials paragraph).

From the perspective of attachment research, Zayas and Shoda (2005) found a relation between Mother-IATs and Partner-IATs and an explicit measure of adult attachment (ECR). Low negative correlations were found between the Mother-IAT scores, defined as an association between the dichotomies “pleasant/unpleasant” and “supportive/rejecting” and the ECR-subscale Avoidance. For the Partner-IAT scores, participants were asked to indicate words that were highly related and highly unrelated to their partners. These words were then used as stimulus words to be classified according to a dichotomy “name of the partner/not-name of the partner.” In this case, the correlation with ECR-Avoidance was negative, but considerably higher. This last finding was consistent with Banse and Kowalick's (2007) comparison between groups of women in different stressful conditions. It seems that positive representations of partners constitutes a resource for coping in stressful life situations. Dewitte et al. (2008, p. 282) used the IAT “as an index of the implicit attachment self-concept” and found that self-esteem and relational anxiety on the IAT were, in fact, correlated with attachment style, and able to predict strategies for successfully managing attachment-related stressful circumstances. The aim of the present study is to extend this work on implicit processes related to attachment. We hypothesize an effect of attachment styles in shaping the implicit representations of caregiving related perceptions. Because of the centrality of attachment in affecting individual development, we propose a role of attachment dependent schemata in structuring the implicit activation determined by parenting behavior.

### Attachment and caregiving systems

The fundamental role of the caregiver in influencing children's attachment behaviors and representations lead to the study of the caregiving behavioral system (George and Solomon, 2008). Bowlby (1969/1982, 1988) proposed the existence of a behavioral structure in caregivers, which is reciprocal to the recipients' attachment behavior. Parenting entails moving from a position characterized by seeking protection and security to one which requires providing protection and care. The connection between the two systems is suggested by their shared evolutionary aim. In fact, Belsky et al. (1991, p. 172) proposed a definition of attachment as an evolved psychological mechanism, through which the parents' experiences during childhood and adolescence are transmitted “probabilistically” to their offspring, shaping their development and reproductive approaches. The strategies (in both behavioral domains and emotion regulation) learnt in infancy, constitute an adaptive advantage because they promote faster and more specific responses within the EEA. As a consequence, such strategies tend to remain relatively stable during the life-span, as confirmed by empirical research (Fraley, 2002; see also Raby et al., 2015, for the transmission of infant attachment). Numerous studies support the strong correspondence between mothers' states of the mind with respect to attachment and their children's attachment (van IJzendoorn, 1995a). This evidence seems stable and constant in different cultures (Grossmann et al., 1988; Sagi et al., 1997; Kazui et al., 2000; Hautamäki et al., 2010) and in different risk populations (Bus and van IJzendoorn, 1997; Tarabulsy et al., 2005; McMahon et al., 2006; Shlafer et al., 2015). In Bowlby's (1969/1982) and Ainsworth's (Ainsworth et al., 1974, 1978) view, the mechanism responsible for this transmission should be the so called “maternal sensitivity,” defined as the ability of being aware of infants' signals and to correctly respond to them. The effect they hypothesized was that an insecure mother's attachment lead to a poor maternal sensitivity and in turn to the transmission of mother's attachment to the infant. Unexpectedly, research identified a “transmission gap” (van IJzendoorn, 1995a,b; van IJzendoorn and Bakermans-Kranenburg, 1997) because the mediating role of maternal sensitivity was significant but modest. Fonagy and Target (2005) proposed the mediating function of mothers' ability to mentalize as a necessary factor for creating a secure base environment for their children, as empirical research confirmed (Grienenberger et al., 2005; Slade et al., 2005). From a developmental perspective, Meins (Meins et al., 2001, 2012) proposed that is mothers' mind-mindedness, i.e., the capacity to understand children's mental states, what enables them to respond to the children's needs adequately. More recently, Bernier et al. (2014) proposed to add the notion of “maternal autonomy support” to describe mothers' ability to enhance children's confidence in exploring the environment. This approach seems to take into account the problem of the transmission gap, although further studies are needed to explore underlying social cognition processes. Scharf and Mayseless (2011) followed 60 men from adolescence to early adulthood and found a continuity between their state of mind with respect to attachment in adolescence and the quality of parenting 9 years later. Other studies used self-reported attachment measures (for reviews, see: Jones et al., 2014, 2015a,b) and found that insecurity is associated with more negative attitudes toward parenting, which is considered more stressful (Nathanson and Manohar, 2012). Secure individuals consider themselves more competent and effective in parenting, compared to less secure ones (Kilmann et al., 2009; Howard, 2010; Caldwell et al., 2011; Kohlhoff and Barnett, 2013). Anxiety was found to be associated to the perception of infants as interfering with parents' romantic relationship (Rholes et al., 2011) and jealousy toward the children (Wilson et al., 2007). Parental avoidance is correlated with less optimistic expectations for child outcomes (Lench et al., 2006).

From an evolutionary perspective, intergenerational transmission seems to foster the maintenance of strategies which are able to enhance the reproduction of the species (Belsky, 2005), but the emotional and cognitive mediators remain unclear. In particular, literature focused primarily on correlational studies, with a less intense focus on experimental manipulations.

### The present study

Although there is a growing theoretical interest about the attachment transmission gap, few studies focused on the cognitive processes that may affect the individual's relational patterns. The aim of this study was to explore the link between adult attachment styles and attitudes toward different caregiving behavioral modalities. In particular, we investigated if current adult attachment styles make individuals discriminate different ways of caregiving, and if they are associated with a preference for a specific caregiving modality. We hypothesize that the preference for proximity, avoidance, or resistance in adult relationships could predict the attitude toward the perception of a new specific relationship (Brumbaugh and Fraley, 2007), like a caregiving one.

From an evolutionary perspective, the intergenerational transmission should foster the maintenance of strategies able to lead to reproduction of the species (Belsky, 2005). Therefore, if these strategies experienced by an individual led to reproduction, there is no need to change them and they can be transmitted to next generations. Then we expect adult attachment styles to shape the adult perception of caregiving strategies.

In order to explore the association between attachment styles and the perception of different caregiving modalities, we designed a study to elicit a specific caregiving representation in the participants and, then, we assessed the attitude to the semantic category “mother.” Our first hypothesis was that the activation of a caregiving representation in line with the participants' attachment style would entail a more positive attitude, implying a preference for a strategy that is coherent with one's own attachment style. For example, a participant who uses dismissing strategies in his/her adult relationships should show a more positive attitude when he/she is watching a dismissing caregiver than when he/she is watching a preoccupied mother and her baby.

Second, a fundamental question relates to whether the transmission of attachment styles is an automatic or implicit process of identification with an experienced caregiving modality. Do individuals have “script-like representations of secure base experiences” (Waters and Waters, 2006, p. 185) and do they use them as basis for comparison in forming attitudes of different situations? In order to face this issue we used two different measures of attitude, an implicit one and an explicit one. We expect a stronger effect of attachment style on the more implicit measure, because of the importance of automatic processes in IWMs.

Third, in the light of the strong connection between attachment styles and emotion regulation strategies, we are interested in linking the perception of different caregiving styles to a measure of emotion arousal. The process of forming attitudes has also an affective component (Petty et al., 2001; Clore and Schnall, 2005; Malhotra, 2005) so we propose at an explorative level an influence of attachment styles on the emotional processes that contribute to define a specific attitude toward a caregiving style. More specifically, disgust seems to be involved in the process of formation of attitudes, for instance in political orientations (Hibbing et al., 2014; Inbar and Pizarro, 2014). Disgust fosters avoidance not only of dangerous or unhealthy situations but also of unfair or not convenient conditions (Chapman et al., 2009; Chapman and Anderson, 2013; Tybur et al., 2013). The evolutionary purpose is to avoid unhealthy or unfamiliar behaviors, so it is possible that the same process takes place while an individual is selecting between different caregiving behaviors. Magai et al. (2000) found an effect of attachment “Preoccupation” on facial expression of disgust during an emotion elicitation task. For exploratory purposes, we want to test if this role of disgust can be extended during the perception of caregiving modalities. Then we tested whether the expression of disgust during the observation of a specific caregiving modality in adults depends on individual's attachment style. A caregiving modality less coherent with participant's attachment style should elicit more disgust. We are interested in this effect as a first step toward a better understanding of the mechanisms to form this kind of attitudes. This effect could be part of the process that increases the chances of intergenerational transmission of attachment style, influencing the formation of attitudes and driving behaviors. Attachment style would be confirmed as crucial in the psychophysiological process of discerning between different caregiving behaviors and the prominence of low-level information processes in forming these attitudes. The attitudes toward caregiving modalities would directly shape the emotions expression and regulation: this would suggest an explanation of transmission gap in a sort of communication at an implicit level between mother and child.

## Materials and methods

### Participants

Seventy-three Italian undergraduate students participated in exchange for course credits at the Psychology Department of the University of Milan-Bicocca. The only exclusion criteria was having children. Twenty-five participants (12 males) were assigned to a preliminary phase; 48 females were assigned to a second step of the present study.

### Materials

#### Experiences in close relationship-revised (ECR-R; Fraley et al., 2000; Busonera et al., 2014)

The ECR-R is a questionnaire used to assess adult romantic attachment style as resulting of two orthogonal dimensions: a subscale of anxiety and a subscale of avoidance. High scores on the first subscale indicate a tendency to preoccupation, jealousy and fear of abandonment, while high scores on the second scale suggest uneasiness with intimacy. The questionnaire is largely used to measure individual differences in romantic adult attachment styles and shows good psychometric properties both for validity and reliability (Sibley et al., 2005; Fairchild, 2006).

#### Single target-implicit association task (ST-IAT; Wigboldus et al., 2004)

The ST-IAT is a version of IAT (Greenwald et al., 1998) that measures the level of association between two categories characterized by opposite polarities (e.g., pleasant/unpleasant) and a single target category (in the present study the category was “mother”). It is a computer task developed with Inquisit 4 (Inquisit, 2013): participants are asked to categorize the words that appear in the center of the screen depending on the category they belong to. By pressing different response keys, they associate these words to the categories presented on the top part of the screen: the left key for the left categories or the right key for the right categories. The words can refer to the a polarity (e.g., “wonderful” vs. “awful”) or to a target category (e.g., “care” or “comfort”). In different trials target category can be presented on the right or on the left, that is can be associated with each of the opposite polarities (“good,” “bad”). The differences in reaction times between the conditions when the category is associated with each of the two polarities represent a measure of association between one side of the polarity and the target category. In our task the IAT algorithm (Greenwald et al., 2003) produced the score of the association between the category “good” and the category “mother.” IAT has been shown to have good predictive validity (Greenwald et al., 2009) and validity of the scoring algorithm (Richetin et al., 2015). It is one of the most used task to study implicit attitudes (Teige-Mocigemba et al., 2010) and it has been used also in attachment research (Zayas and Shoda, 2005). The words used in the present task are the following. Positive: Marvelous, Superb, Pleasure, Beautiful, Joyful, Glorious, Lovely, Wonderful. Negative: Tragic, Horrible, Agony, Painful, Terrible, Awful, Humiliate, Nasty. Mother: Care, Attention, Consolation, Support, Help, Bond, Comfort.

#### Semantic differential (SD; Osgood et al., 1957)

Semantic differential is an explicit measure of attitude. The participant is asked to think to a category (in our study “mother”) and to rate 10 bipolar adjective on 7-point Likert scales (e.g., “strong/weak,” “good/bad”). It has been recently used to evaluate different kinds of parents (Weed and Nicholson, 2015), showing a good discriminant ability.

#### FaceReader software

*FaceReader software* version 5.0 (FR, Noldus, 2013) automatically analyzed facial expressions to detect the six basic emotions described by Ekman (1992): happy, sad, angry, surprised, scared, disgusted and a neutral state. The software showed a good convergent validity (Den Uyl and Van Kuilenburg, 2005; Terzis et al., 2012) with the manually coded FACS ratings (Ekman and Rosenberg, 1997). FR reduces the time for behavioral coding without compromising accuracy and its use in psychological studies is increasing (Chentsova-Dutton and Tsai, 2010; He et al., 2014).

#### Stimulus materials

Three different videos of mother–infant interactions were employed to activate three different representations of caregiving. The videos were chosen based on mothers' Adult Attachment Interview (AAI; George et al., 1985) classification, independently obtained. We selected the videos from a sample of mother infant free play (with standardized games) used in a previous study (Tagini et al., in preparation). The children in the selected videos were females of 23 months of age. Mothers' AAI classification was previously assessed and the mothers were selected to be one secure, one preoccupied and one dismissing. In addition, a further selection criteria was that all ECR-R (which was assessed as well) scores of mothers were consistent with their AAI classification. The secure mother had low anxiety (less than a standard deviation from the mean) and low avoidant scores, the preoccupied one was high in anxiety (more than a standard deviation from the mean) and the dismissing one was high on avoidance. After watching the three videos, 18 attachment experts (researchers and clinicians) answered the following questions for each video: “How much do you consider the caregiving behavior of this mother prototypic of a secure/preoccupied/dismissing mother? One is not prototypic at all and seven is very prototypic.” Results confirm that our stimuli has been chosen to be actually able to represent different caregiving styles and this association seems to be empirically supported.

### Procedure

This study was carried out in accordance with the recommendations of the Declaration oh Helsinki and the approval of the Ethical Committee of University of Milano—Bicocca. All subjects gave written informed consent in order to participate.

The first phase of the experiment consisted in evaluating the association between measures of attitude and participants' attachment style. The implicit and explicit measures were assessed for a first sample of 25 participants (females = 14) and subsequently their attachment style was evaluated with the ECR-R. Because no significant correlations were found, the presence of an effect of attachment style on attitudes during the second phase, could be interpreted a consequence of the experimental manipulation. The second phase consisted in a new, larger sample of female participants (*N* = 48), who watched the videos of mother-child interactions. In each session, after viewing the mother-child interaction video, the participants' attitude toward the category “mother” was measured, by means of both implicit (ST-IAT) and explicit (SD) measures. Thus, differences in attitudes could be attributed to having watched the different videos. The order of the videos was randomly generated to control learning effects on the ST-IAT. Three days was the minimum interval between one condition and the subsequent one. The choice to select only female participants was due to the characteristics of the videos, that represent mothers playing whit their daughters. This could make very difficult to interpret any possible gender difference in the data.

At the end of the first session, each participant was asked to fill out the ECR-R. The number of observations (*N* = 138) is slightly less than the maximum possible number (*N* = 144), because not all participants came three times to perform the task in the three different conditions. Participants who came just once were excluded from the analysis (*N* = 4).

While participants were watching the videos, their faces were video recorded in order to analyze their emotional expressions.

### Data analysis

We used R software (R Development Core Team, 2013) to analyze data. Correlations between the attachment measure and the ST-IAT and SD in the condition when no caregiving representation was activated (no video watched) were calculated, in order to control the possibility that the attitude toward the category “mother” depended on attachment style.

The effects of the experimental manipulation on ST-IAT and SD was tested with linear mixed models (LMMs). This allowed us to test within subjects experimental effects, considering attachment measures as covariates, and to perform repeated measure analyses, including those participants who were evaluated in only two conditions. The fixed effects were attachment style (anxiety and avoidance of attachment) and the type of condition (the vision of a video with a preoccupied/secure/dismissing mother). The outcome variable was the attitude toward the category “mother,” in one model it was the explicit measure (SD), and in the other model the implicit one (ST-IAT). We report all significant main effects and interactions (up to three way interactions).

The same predictors were used in a different model, with the Disgust measure of FaceReader Software as the outcome variable. Because of the very skewed distribution (skewness = 3.27 and kurtosis = 11.97) the outcome measure was recoded as a binomial variable (median split), in which 0 indicated a low Disgust mean and 1 a high Disgust mean. In this way, we could test a logistic mixed regression model, as part of the generalized linear mixed models (GLMMs).

Linear mixed models and GLMM were performed in R by using the package “lme4” (Bates et al., 2015) and bootstrapping all the confidence intervals. Degrees of freedom and *p*-values for the LMMs were computed via Kenward–Roger's approximations (*F*-tests) and Satterthwaite's approximations (*t*-tests) through the “lmerTest” package (Kuznetsova et al., 2014). The GLMM p values and degrees of freedom for the Chi square tests were computed via the likelihood ratio tests through the package “afex” (Singmann et al., 2014). Plots were built using the package “ggplot2” (Wickham, 2009).

## Results

### Correlation between attachment style and attitude measures without manipulation

The first step was to test the correlation between the two dimensions of attachment style (avoidance, anxiety) and the two measures of attitude (ST-IAT, and SD), when no representation of caregiving was activated. This analysis was performed on the first sample (*N* = 25) that did not watch any video. The results indicate that there was no significant association, both for the ST-IAT (avoidance: *r* = 0.09; anxiety: *r* = −0.06) and for the SD (avoidance: *r* = −0.13; anxiety: *r* = −0.16). The non-significant correlations obtained allowed us to ignore the “baseline” condition (no experimental manipulation) in subsequent sample, in which it was therefore possible to consider the effect of attachment style as related to the experimental manipulation.

### Effects of attachment style and conditions on IAT

**Figure 1:**
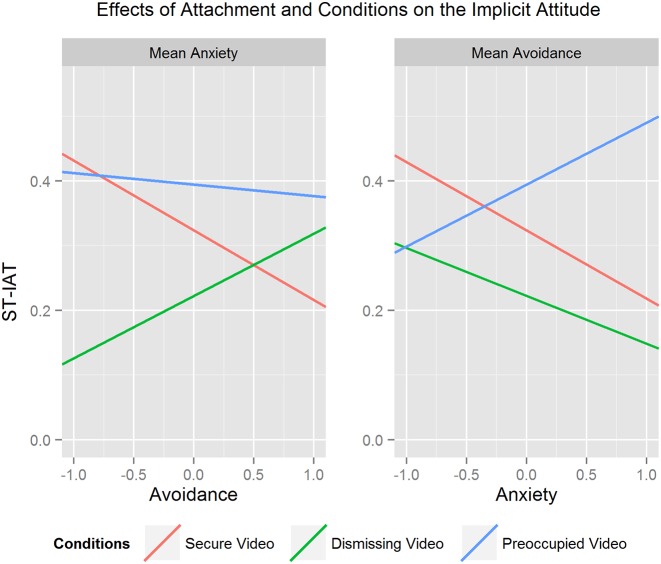
**Results of the simple slope analysis of the ST-IAT model: effects of attachment styles and conditions**. The left panel shows the effects of avoidance when Anxiety is centered on the mean, while the right panel refers to anxiety effects when Avoidance is centered on the mean.

The first LMM model (within-subjects factor: type of conditions; covariates: anxiety and avoidance of attachment, centered on the means) showed no effect of the predictors (neither main effects nor interaction effects), on the attitude measured in an explicit way. The random terms were both intercept (var = 0.11) and condition (var = 0.26), because the model with both effects showed a significant improvement of fit [ΔBIC = −44.48, χ^2^(5) = 54.48, *p* < 0.001]. The same LMM model was performed with the ST-IAT scores as the outcome variable. The random term was just the intercept (var = 0.04), because the model with intercept and condition effects showed a not significant improvement of fit [ΔBIC = −17.89, χ^2^(5) = 6.71, *p* = 0.24]. IAT score was significantly affected by the type of Conditions [*F*(2, 83.20) = 5.83, *p* < 0.01] but not by Avoidance [*F*(1, 42.90) = 0.06, *p* = 0.80] and Anxiety [*F*(1, 43.41) = 0.45, *p* = 0.51]. The three two-ways interaction terms were the following: Conditions × Avoidance, *F*(2, 82.32) = 8.53, *p* < 0.001; Conditions × Anxiety, *F*(2, 82.82) = 7.86, *p* < 0.001; Avoidance × Anxiety *F*(1, 42.80) = 0.26, *p* = 0.61. The three-way interaction Condition × Anxiety × Avoidance was significant, *F*(2, 82.22) = 3.85, *p* < 0.05, suggesting that the effect of type of conditions (one of the three videos) was different for different levels of the two attachment style scales. To interpret this interaction we calculated the simple slope analysis: on the left panel of Figure 1 the effects of Avoidance are shown (Anxiety centered on the mean), while on the right panel of Figure 1 the effects of Anxiety are shown (Avoidance centered on the mean).

When anxiety is centered on the mean, avoidance has a negative effect on implicit attitude after the Secure Video, *b* = −0.11, SE = 0.05, *t*(89.83) = −2.26, *p* < 0.05, 95% CI [−0.20, −0.004], a positive effect after the Dismissing Video, *b* = 0.10, SE = 0.05, *t*(90.54) = 2.02, *p* < 0.05, 95% CI [0.004, 0.189] and a negative not significant effect after the Preoccupied Video, *b* = −0.02, SE = 0.05, *t*(90.48) = −0.37, *p* = 0.71, 95% CI [−0.11, 0.81].

When avoidance is centered on the mean, anxiety has a negative effect on implicit attitude after the Secure Video, *b* = −0.10, SE = 0.05, *t*(92.77) = −2.00, *p* = 0.05, 95% CI [−0.21, −0.001], a negative not significant effect after the Dismissing Video, *b* = −0.07, SE = 0.05, *t*(90.85) = −1.42, *p* = 0.15, 95% CI [−0.17, 0.04] and a positive not significant effect after the Preoccupied Video, *b* = 0.09, SE = 0.05, *t*(90.64) = 1.84, *p* = 0.06, 95% CI [−0.0009, 0.01988].

To sum up, we found a positive effect of avoidance on IAT after the dismissing video and negative effects of both anxiety and avoidance after the secure video.

### Effects of attachment style and conditions on emotion expression

There was no significant association between Disgust variable and the explicit or implicit measures of attitude.

**Figure 2:**
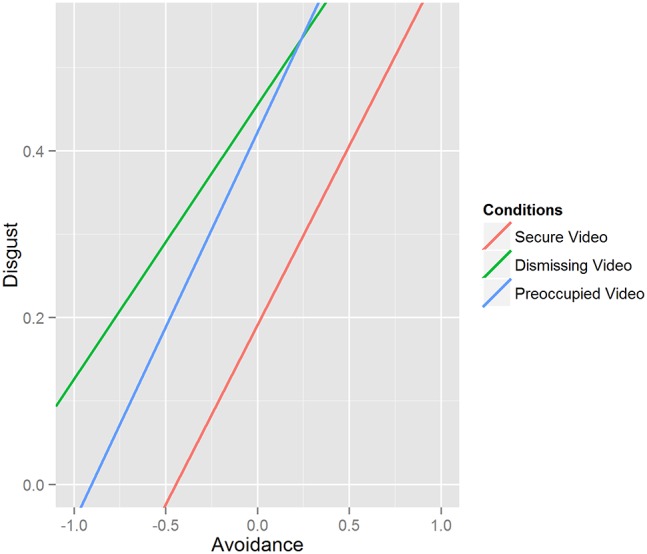
**Results of the simple slope analysis of the Disgust model: effects of attachment styles and conditions**.

A third model with the same predictors and random effects was performed with the FaceReader measure of disgust as dichotomous outcome variable. The random term was just the intercept (var = 0.00) because the model with intercept and condition effects was not able to converge. The results showed a main effect of avoidance [χ^2^(13) = 4.46, *p* = 0.03] and no other significant main or interaction effect. The effect is positive, as it can be seen in Figure 2 that represents the effect of the three different conditions even if no one of them is significant if taken singularly. Secure Video: *b* = 0.43, SE = 0.36, *z* = 1.19, *p* = 0.23, 95% CI [−0.25, 1.20]; Dismissing Video: *b* = 0.33, SE = 0.31, *z* = 1.04, *p* = 0.30, 95% CI [−0.27, 0.99]; Preoccupied Video: *b* = 0.47, SE = 0.33, *z* = 1.42, *p* = 0.15, 95% CI [−0.14, 1.16].

## Discussion

The results of this study confirmed the adequacy of the experimental paradigm in activating a specific caregiving representation, and measuring the process of forming an attitude. We found a role of adult attachment style in shaping the implicit attitude, but non the explicit one, toward the category “mother.” The explicit attitude seems not to be influenced neither by the manipulation neither by participants' attachment style. This can be due to social desirability because the perception of the category “mother” is highly expected to be always good, or also to some kind of inability in being aware of a preference for a relational modality. On the contrary, the significance of the implicit attitude model could suggest the importance of the automatic processes of the IWMs. Participants are able to compare their relational expectations with the behavior they see in the videos, but they process this information in a not conscious way.

The results of the implicit attitude model are consistent with our expectations. In fact both Avoidance and Anxiety have a negative effects on IAT scores after the Secure Video (when the other variable is centered on the mean). This means that the more insecure a participant, the more negative her attitude toward the secure mother. On the contrary, avoidance has a positive effect on IAT scores after the Dismissing Video, but no effect after the Preoccupied one. This seems consistent with our hypotheses: very avoidant participants tend to prefer the Dismissing Video, while we had no predictions of a role of Avoidance on implicit attitude after the Preoccupied Video. Anxiety plays a similar role because it has a positive marginally significant effect on attitude after the Preoccupied Video and a negative not significant effect after the Dismissing Video.

The link between adult attachment style and the perception of different ways of caregiving can contribute to a better understanding of the mechanisms underlying the transmission of attachment. Avoidance or seeking of proximity in adult attachment seem to impact implicit processes of evaluation of caregiving modalities. The continuity between representations of partners and responses in new social encounters (Brumbaugh and Fraley, 2007) has a specific feature for what concerns caregiving relationships. From an evolutionary perspective, attachment schemata are influenced by the caregiving style of the parents. Thus, if an individual reaches the goal of reproduction, it is implied that the caregiving strategies have succeeded in that specific environment. In this sense it seems inexpensive to maintain a continuity between generations. It is consistent with the definition of IWMs that they regulate the access to information relevant to attachment, and our findings underline that the information relevant for the caregiving system is also closely related to attachment styles.

The explorative hypothesis of the effect of the experimental manipulation and attachment style on the facial expression of disgust while participants were watching the different videos was not confirmed. The only significant effect was a positive one of avoidance that was also unexpected because Magai et al. (2000) found an association between facial expression of disgust and “Preoccupation” during an emotion elicitation task. Anyway it seems reasonable that a specific kind of human relationship like the mother infant one elicits more negative physiological reaction in participant with higher levels of avoidance, although this was not our hypothesis. Finally, we found no association between disgust and the two measures of attitude, so we can claim that the process of forming attitudes is not explained by the elicitation of this specific emotion.

## Conclusion

Adult attachment styles seem to play a moderating role on high level implicit attitudes toward caregiving but not on explicit attitudes or on low level regulation and expression of emotion. These findings underline the fundamental role of adult attachment style in predicting attitudes related to the caregiving system. Our hypotheses are only partially confirmed because we found a correspondence only between attachment styles and the IAT. Anyway, this specific result is in line with Bowlby's statements on the complementarity of the caregiving and attachment motivational systems. Furthermore, our study confirms his belief in the complexity of the effects involved. In fact, Bowlby (1988) wrote: “Parenting behavior, as I see it, has strong biological roots, which accounts for the very strong emotions associated with it; but the detailed form that the behavior takes in each of us turns on our experiences – experiences during childhood especially, experiences during adolescence, experiences before and during marriage, and experiences with each individual child” (p. 5). From a clinical point of view, these issues are of great interest since they may contribute to the process of the intergenerational transmission of attachment, and the passing on of disorders, considering that an insecure attachment style can become a risk factor for psychopathology (Mikulincer and Shaver, 2012). It has in fact been associated to internalizing and externalizing problems in adolescence (Sarracino et al., 2011) clinical disorders such as depression (Roberts et al., 1996; Cantazaro and Wei, 2010; Santona et al., 2015), anxiety disorders (Warren et al., 1997), and personality disorders (Meyer and Pilkonis, 2005; Crawford et al., 2007).

The limitations of this study must be acknowledged. In fact, research on adult attachment reports a “trivial to small” correspondence between adult attachment style questionnaires and state of mind with respect to attachment (Roisman et al., 2007). Then a limitation of our study is the use of different models of measure between stimuli and participants' assessment. AAI is indeed considered the “gold standard” for adult attachment assessment (Hesse, 2008) and differs from adult attachment styles questionnaires because of its implicit nature. AAI relies on different processes and measures coherence of mind with respect to attachment, which, by definition, differs from the explicit thoughts about attachment style. Finally the correspondence between an individual's attachment style and his preference for the matching caregiving modality could however not be directly tested, because of the attachment model underlying the measurement used, which differed from the stimulus categorization. On one hand the continuous measure of attachment is more effective (Fraley et al., 2015), on the other hand led to a more difficult interpretation of the results because the videos had to be categorized.

A future perspective could explore the moderating role of gender in evaluating caregiving representations. Although differences in attachment style usually do not emerge from studies based on AAI (Bakermans-Kranenburg and van IJzendoorn, 2009), it seems reasonable that males and females could have different attitudes toward caregiving behaviors. In an evolutionary view, males tend to be more facultative investors (Del Giudice, 2009) than females, probably because a low parental investment can be more adaptive in order to save resources for additional mating. Thus, regardless of the effect of attachment style, we could expected higher levels of positive attitudes when representations were activated in females, due to evolutionary differences.

Approaching the transmission gap issue, the evidence of the non-linear effect of maternal sensitivity in shaping children attachment led to focusing on the role of other mediating constructs, such as Reflective Function (Fonagy and Target, 1997; Slade et al., 2005). This study attempted to investigate, at a more basic level, the link between attachment style and caregiving representations and we found an effect that may be a starting point for further research and for interventions on parenting skills in risk situations. Our results confirm that cognition and behavior linked to caregiving rely on automatic processes (Bowlby, 1969/1982; Soltis, 2004; Bos et al., 2010; Riem et al., 2011; Swain et al., 2014). In this regard, a key research area may be the study of the maternal representations, in order to understand how this caregiving attitude emerge and develop in pregnancy and early motherhood (Stern, 1995; Innamorati et al., 2010). It seems that every kind of intervention should face these implicit processes that do not reach a conscious level of elaboration. Finally, the results may be relevant to psychotherapy, considering that the interconnection between representations and the perception of relational contexts is a key concept in those contexts that focus on pointing out specific non-adaptive representations and reflecting on which specific environment allowed its development.

### Conflict of interest statement

The authors declare that the research was conducted in the absence of any commercial or financial relationships that could be construed as a potential conflict of interest.
